# Uptake of external cephalic version for term breech presentation: an Australian population study, 2002–2012

**DOI:** 10.1186/s12884-017-1430-5

**Published:** 2017-07-26

**Authors:** Yu Sun Bin, Christine L. Roberts, Michael C. Nicholl, Jane B. Ford

**Affiliations:** 1 Clinical and Population Perinatal Health Research, The Kolling Institute, University of Sydney, Building 52, Royal North Shore Hospital, St Leonards, NSW 2065 Australia; 2 Sydney Medical School Northern, University of Sydney, Sydney, NSW Australia; 3 Department of Obstetrics and Gynaecology, Royal North Shore Hospital, St Leonards, NSW Australia

**Keywords:** Prenatal care, Breech presentation, Term birth, External cephalic version, Retrospective studies, Australia

## Abstract

**Background:**

The safety, efficacy, and cost-effectiveness of external cephalic version (ECV) for term breech presentation has been demonstrated. Clinical guidelines recommend ECV for all eligible women, but the uptake of this procedure in the Australian healthcare setting is unknown. This study aimed to describe ECV uptake in New South Wales, the most populous state of Australia, during 2002 to 2012.

**Methods:**

Data from routine hospital and birth records were used to identify ECVs conducted at ≥36 weeks’ gestation. Women with ECV were compared to women who were potentially eligible for but did not have ECV. Eligibility for ECV was based on clinical guidelines. For those with ECV, birth outcomes following successful and unsuccessful procedures were examined.

**Results:**

In *N* = 32,321 singleton breech pregnancies, 10.5% had ECV, 22.3% were ineligible, and 67.2% were potentially eligible but did not undergo ECV. Compared to women who were eligible but who did not attempt ECV, those who had ECV were more likely to be older, multiparous, overseas-born, public patients at delivery, and to deliver in tertiary hospitals in urban areas (*p* < 0.01). Fewer women who underwent ECV smoked during pregnancy, fewer were morbidly obese, and fewer had a hypertensive disorder of pregnancy, compared to those who were eligible. Caesarean section occurred in 25.9% of successful compared to 95.6% of unsuccessful ECVs. Infant outcomes did not differ by ECV success.

**Conclusions:**

The majority of women with a breech presentation did not receive ECV. It is unclear whether this is attributable to issues with service provision or low acceptability among women. Policies to improve access to and information about ECV appear necessary to improve uptake among women with term breech presentation. Improved data collection around the diagnosis of breech presentation, ECV attempts, and outcomes may help to identify specific barriers to ECV uptake.

## Background

Breech presentation refers to fetuses that lie bottom- or feet- first rather than head-first. External cephalic version (ECV) is an effective manipulative procedure for turning a breech-presenting fetus so that it presents head-first for vaginal delivery [1, 2]. Adverse outcomes associated with ECV are rare [3] and the procedure is cost-effective when compared to a scheduled caesarean section [4].

Australian and international clinical guidelines recommend ECV at or near term (37+ weeks gestation) for all women with uncomplicated breech presentations where there are facilities for an emergency caesarean section [5, 6]. It is unknown how well these recommendations have been translated into practice. Thus the aim of this study was to describe the uptake of ECV in the Australian healthcare setting and the maternal and pregnancy characteristics associated with ECV uptake.

## Methods

### Data sources

New South Wales (NSW) is the most populous state in Australia with approximately 93,000 births each year [7]. Data for this study came from four routine population health datasets in NSW: the Admitted Patient Data Collection (hospital records), the Perinatal Data Collection (birth records), the Perinatal Death Reviews database and the Register of Births, Deaths, and Marriages (death records). The hospital records are a census of discharges, transfers, and deaths in NSW public and private hospitals and day procedure centres. Diagnoses and procedures associated with each hospital record are coded according to the International Statistical Classification of Diseases and Related Health Problems (ICD-10-AM) [8] and the Australian Classification of Health Interventions [9]. The birth records describe all births in NSW of at least 20 weeks gestation or at least 400 g birth weight and are completed by an attending midwife or medical practitioner who records information on maternal health, pregnancy, labour, delivery, and infant characteristics. The state-mandated Perinatal Death Reviews and the Register of Deaths were used to confirm deaths recorded in the hospital and birth records.

The datasets were linked by the NSW Centre for Health Record Linkage using probabilistic record linkage [10]. To preserve privacy, personal identifiers were removed and a linkage key was provided to researchers so that records could be merged for the current study. Ethics approval for the record linkage and for conducting the study was obtained from the NSW Population Health Services Research Ethics Committee.

These population datasets have been validated: conditions such as pregnancy diabetes and hypertension are coded using a combination of birth and hospital records and are comparable to medical records [11, 12]; delivery characteristics and other medical conditions identified using these records have been shown to be highly accurate when compared to medical record review [13–16].

### Study population

**Fig. 1:**
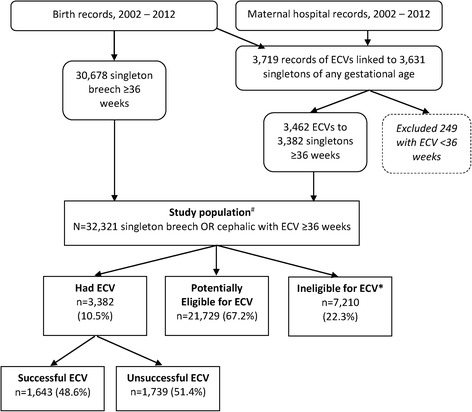
Selection of study population from available records and definition of the study groups. *Ineligible for ECV for any of the following reasons (number excluded; % of total ineligible): oligohydramnios (580; 8.0%), previous CS (5273; 73.1%), pelvic abnormality (352; 4.9%), placenta praevia (483; 6.7%), placenta accreta (124; 1.7%), antepartum haemorrhage or abruption (651; 9.0%), infant has major congenital anomaly (466; 6.5%)

The study population included all women with singleton breech pregnancies at or near term (≥36 weeks) during the 11-year period 1st January 2002 to 31st December 2012. Due to the data structure, the study population was derived by combining: [1] women who had a record of ECV at ≥36 weeks during pregnancy, and [2] breech-presenting singleton infants born at ≥36 weeks, taken from birth records (Fig. 1). To examine the uptake of ECV, the population of breech pregnancies were classified retrospectively into 3 groups: [1] women with ECV, [2] women potentially eligible for ECV, and [3] women considered ineligible for ECV.

**(1) Women with ECV:** Women were considered to have undergone ECV if procedure code “16,501 External cephalic version” was found in maternal hospital records during pregnancy and a corresponding infant birth record could be identified. We estimated gestational age at ECV using gestational age from the birth record and the date of ECV from the hospital record. If women had more than one pregnancy during the 11-year study period, all pregnancies were included in the study population. For women with multiple ECVs during the same pregnancy, only the last ECV was described.

**(2) Women Eligible for ECV:** We categorised women without ECV into those potentially eligible or potentially ineligible for ECV. This retrospective classification was based upon clinical guidelines from the Royal Australian and New Zealand College of Obstetricians and Gynaecologists [5]. Women were considered potentially eligible for ECV if singleton birth was at or near term (≥36 weeks) and if none of the following were recorded in the birth or hospital records: oligohydramnios, antepartum haemorrhage or abruption, previous caesarean section or pelvic abnormality, placenta praevia, placenta accreta, and an infant with major congenital anomalies.

**(3) Women ineligible for ECV:** Women were considered ineligible for ECV if they had any of the absolute contraindications to ECV listed above.

To examine the maternal and pregnancy characteristics associated with ECV, sociodemographic and health characteristics were taken the hospital and birth records. Maternal hypertension and diabetes were identified according to previously validated algorithms [11, 12]. Patient and hospital type were coded according to previously established categories [17].

For women who received ECV, birth outcomes for infants were compared between those with successful and unsuccessful ECVs. Since the outcome of ECV was not recorded, we defined ‘successful ECV’ as cephalic presentation at delivery and ‘unsuccessful ECV’ as breech presentation at delivery. We acknowledge that these definitions may misclassify a small proportion of fetuses which revert to breech after a successful procedure or spontaneously turn to cephalic presentation after a unsuccessful procedure.

For those women who underwent ECV, we examined birth outcomes by ECV success. These outcomes included gestational age at birth, mode of delivery, perinatal death (fetal and neonatal deaths), neonatal intensive care or special care nursery (NICU/SCN) admission, and Apgar scores <4 at 1 min and <7 at 5 min. All infant outcomes were recorded on the birth record, except perinatal death which also took into account the hospital and death records.

### Statistical analysis

Characteristics of women who underwent ECV and those potentially eligible and ineligible for ECV were tabulated. Since we were mainly interested in women who underwent ECV compared to those potentially eligible for but without ECV, only these groups were directly compared using chi-square tests for categorical variables and t-tests for continuous variables. Birth outcomes following ECV were tabulated according to ECV success. Differences between the groups were examined using chi-square tests or Fisher’s exact test for rare outcomes. There was minimal missing data (<0.1%). All analyses were conducted using SAS 9.3 (SAS Institute, NC).

## Results

Of 32,321 singleton breech pregnancies, 10.5% had a record of ECV at or near term, 22.3% were considered ineligible for ECV, and the remaining 67.2% were potentially eligible for but did not undergo ECV (Fig. 1). Of those considered ineligible for ECV, the most common reason appeared to be previous caesarean section (73.1%), followed by antepartum haemorrhage or abruption (9.0%), oligohydramnios (8.0%), placenta praevia (6.7%), major infant anomaly (6.5%), pelvic abnormality (4.9%), and placenta accreta (1.7%).

Table 1 compares the characteristics of women who had ECV to those women potentially eligible for but without ECV, as well as women ineligible for ECV. Nearly half of the women who underwent ECV were had previously given birth (46.2%) compared to only a third of those without a record of ECV (32.5%). Women who had ECV were significantly older and more likely to be overseas-born than women without ECV. Women with ECV had lower rates of smoking, morbid obesity, and hypertensive disorders than women who were eligible but did not have ECV. The two groups did not differ on the rate of diabetes.

**Table 1:** Characteristics of women (1) with ECV at or near term, (2) without ECV but eligible for ECV, and (3) ineligible for ECV (*N* = 32,321)

Characteristic	ECV at or near Term *n* = 3382	No ECV but Eligible *n* = 21,729	No ECV and Ineligible *n* = 7210	Difference between ECV and Eligible groups
	n(col%)	n(col%)	n(col%)	Test statistic, *p*-value
Gestational age at ECV				-
36 weeks	1055 (31.2)	-	-	
37 weeks	1239 (36.6)			
38 weeks	646 (19.1)			
39 weeks	266 (7.9)			
≥ 40 weeks	176 (5.2)			
Maternal age (mean, SD)	31.6 (5.1)	30.5 (5.4)	32.5 (5.2)	t(4637.8) = 11.56, *p* < 0.01
≤ 20 years	50 (1.5)	652 (3.0)	71 (1.0)	X^2^ (3)=88.60, *p* < 0.01
20–35 years	2312 (68.4)	15,976 (73.5)	4468 (62.0)	
≥ 35 years	1019 (30.1)	5096 (23.5)	2670 (37.0)	
Parity				X^2^ (1)=235.12, *p* < 0.01
Nulliparous	1819 (53.8)	14,600 (67.3)	1219 (16.9)	
Multiparous	1562 (46.2)	7100 (32.7)	5990 (83.1)	
Country of birth				X^2^ (1)=128.15, *p* < 0.01
Australia	2052 (60.7)	15,286 (70.4)	5067 (70.3)	
Elsewhere	1330 (39.3)	6443 (29.7)	2143 (29.7)	
Smoking during pregnancy	278 (8.2)	2497 (11.5)	882 (12.2)	X^2^ (1)=31.87, *p* < 0.01
Morbid obesity	14 (0.4)	189 (0.9)	135 (1.9)	X^2^ (1)=7.58, *p* < 0.01
Hypertensive disorder	198 (5.9)	2075 (9.6)	1019 (14.1)	X^2^ (1)=48.53, *p* < 0.01
Diabetes	257 (7.6)	1627 (7.5)	834 (11.6)	X^2^ (2)=0.05, *p* = 0.82
Patient type at delivery				X^2^ (2)=239.15, *p* < 0.01
Private	623 (18.4)	6543 (30.1)	2174 (30.2)	
Private patient in public hospital	132 (3.9)	1246 (5.7)	475 (6.6)	
Public	2627 (77.7)	13,940 (64.2)	4561 (63.3)	
Delivery hospital				X^2^ (2)=725.25, *p* < 0.01
Tertiary hospital in Urban area	1550 (45.8)	5520 (25.4)	2033 (28.2)	
Non-tertiary hospital in Urban area	1524 (45.1)	11,216 (51.6)	3584 (49.7)	
Non-tertiary hospital in Regional area	308 (9.1)	4993 (23.0)	1593 (22.1)	

Note: Percentages may not add exactly to 100% due to <0.1% missing data

ECV was strongly associated with hospital and patient type: 77.7% of women with ECV delivered in a public hospital as a public patient compared to 64.0% of those without ECV; and 45.8% of women who had ECV delivered at an urban tertiary hospital whereas only 25.1% of those without ECV did so.

### ECV attempts and success

Of women who underwent ECV, the majority (97.5%) had only 1 attempt; but a maximum of three attempts in the same pregnancy was recorded. A median of 11.0 days (IQR 4–17 days) lapsed between date of (last) ECV and delivery. Three-quarters of women (75.4%) underwent ECV as public patients. Based on fetal presentation at delivery, the success rate for ECV was 48.6% overall: 36.5% in nulliparous and 62.7% in multiparous women.

### Birth outcomes following ECV

Vaginal birth was achieved by three-quarters (74.1%) of those with successful ECV, whilst persistent breech were delivered mainly by caesarean section (95.6%) (Table 2). Earlier gestational age at birth was more common for persistent breech presentation than for successful ECVs, consistent with the high rate of caesarean section in those with unsuccessful ECV. The rates of perinatal death, NICU/SCN admissions, low 1-min Apgar, low 5-min Apgar, and NICU/SCN admissions did not differ significantly between the successful and unsuccessful ECV groups.

**Table 2:** Birth outcomes following ECV, by presentation at delivery (*N* = 3382)

Outcome	Successful ECV (Cephalic at birth) *n* = 1643 n (col%)	Unsuccessful ECV (Breech at birth) *n* = 1739 n (col%)	Test of difference between groups Test statistic, *p*-value
Mode of delivery			X^2^ (2)=1757.48, *p* < 0.01
Vaginal birth	1217 (74.1)	76 (4.4)	
Caesarean section with labour	109 (6.6)	241 (13.9)	
Caesarean section, no labour	317 (19.3)	1422 (81.8)	
Gestational age at birth			X^2^ (5)=697.74, *p* < 0.01
36 weeks	None	6 (0.4)	
37 weeks	62 (3.8)	74 (4.3)	
38 weeks	188 (11.4)	450 (25.9)	
39 weeks	465 (28.3)	953 (54.8)	
40 weeks	514 (31.3)	206 (11.9)	
≥ 41 weeks	414 (25.2)	50 (2.9)	
Perinatal death	8 (0.5)	x (0.2)	Fisher’s exact, *p* = 0.14
Apgar < 4 at 1 min	46 (2.8)	39 (2.2)	X^2^ (1)=1.07, *p* = 0.30
Apgar < 7 at 5 min	30 (1.8)	25 (1.4)	X^2^ (1)=0.80, *p* = 0.37
NICU/SCN admission	138 (8.4)	116 (6.7)	X^2^ (1)=3.63, *p* = 0.06

Note: Cell sizes <5 have been censored

## Discussion

During the 11-year study period, only 10.5% of women with breech presentation at or near term underwent ECV in NSW, with potentially 6 times more women eligible for ECV who did not undergo the procedure.

To our knowledge, this is the first population-based study of ECV uptake in Australia and it shows there is considerable room for improvement in the uptake of ECV. Despite local guidelines suggesting ECV for in all women with uncomplicated breech presentations, these recommendations do not appear to have translated well into clinical practice in the state of New South Wales, Australia’s most populous state.

While ECV has become more common across Australia in the last decade, based on the absolute number of procedures billed to the federally funded healthcare system [18], the uptake of ECV services relative to the number of eligible women with term breech pregnancies is low. Our estimate for the uptake of ECV was 10.5% for all women with breech presentation at or near term, or 13.5% of women considered eligible for ECV. Previous Australian studies have found ECV attempted in 39% of women with breech presentation between 36 and 38 weeks in one hospital [19] and 71% of eligible women in another [20]. The higher rates of uptake in previous studies are unsurprising given that they were conducted in tertiary centres with specialist breech services. This suggests that the provision of specialist breech clinics may facilitate the uptake of ECVs as an effective and low-cost procedure.

Contributors to the low rate of ECV uptake include selection of women for ECV based on criteria other than those stipulated in clinical guidelines and undiagnosed breech presentation. We found a lower rate of morbid obesity and hypertensive disorders in women who had ECV compared to those potentially eligible for ECV suggesting that clinicians are selectively offering ECV to low-risk women, counter to current guidelines for best practice. Previous caesarean section was the most common reason women were deemed ineligible for ECV with only 2.6% of women with ECV having a history of caesarean section compared to 16.6% in women with breech presentation overall. There may be a clinical perception that with a previous caesarean section there are no safe options, however, there is little evidence that caesarean section should be considered an absolute contraindication for ECV [21]. To the contrary, there is some evidence suggesting the success and associated risks of ECV in women with one previous caesarean section are similar to those in women without such history [22, 23]. Similarly, selection on factors favourable to ECV may have occurred: multiparous women were more likely to undergo ECV even though nulliparous women comprise the majority of women with breech presentation. This may also reflect different likelihoods of accepting ECV among nulliparous and multiparous women, especially if women are counselled that success rates are higher in multiparae. This is concerning given the high risk of recurrence for breech presentation [24]; a lower rate of ECV uptake in nulliparous women has implications for not only the first, but also subsequent pregnancies.

Other potential barriers to ECV uptake may include ECV not being offered by clinicians or being declined by women, but the role of these factors are unclear since no population data are collected at these crucial time points. The uptake of ECV was strongly associated with the type of hospital attended by women and public/private patient status suggesting clinicians and women in private and regional hospitals need to be the targets of any intervention to promote ECV uptake.

A study in a large British maternity unit has found that ECV counselling, referral, and attempt rates have increased over recent years, and that intrapartum diagnosis of breech presentation remained the largest barrier to ECV uptake [25]. We suspect that trends in Australia are similar and that intrapartum diagnosis of breech presentation remains a considerable barrier to ECV uptake. We do not know how many undiagnosed breech presentations were included in the current study, but a previous study in an Australian tertiary hospital suggests intrapartum diagnosis occurred in almost 20% of cases [20]. Importantly however, even if this rate of intrapartum diagnosis held true for our study population, a further 40% of breech pregnancies remain eligible for ECV.

The success of ECV in the current study was 37% for nulliparous and 63% for multiparous women and these rates are consistent with those previously reported in Australian hospitals [19, 20, 26, 27]. The overall success rate of 49% was lower than the 60% success rate reported in reviews of the literature [1, 2], but probably reflects lower success in the general population of pregnant women compared to selected trial participants or in specialist clinics with more experienced providers. The relative reduction in caesarean section was similar: a quarter of women with successful ECV delivered via caesarean section compared to 21% in other studies [2].

We found that neonatal outcomes were similar regardless of ECV success, thus providing a stronger incentive for attempting ECV in conjunction with existing evidence in the Australian setting [19, 20, 26, 27], and world-wide, that the complications of ECV are rare and unrelated to the success of ECV [3]. The small risks of serious complications (<0.4%) leading to emergency caesarean section [3] must be weighed against the substantial potential benefits of a successful ECV and the advantages of avoiding a caesarean section or vaginal breech delivery.

### Strengths and limitations

The strength of this study is the use of large, reliably reported, population data for describing the characteristics and outcomes associated with ECV in Australia. Limitations of the study include potential under-ascertainment of ECV procedures and some contraindications for ECV. We found a low rate of ECV uptake and this might reflect some under-ascertainment of ECV procedures. ECVs may be performed as outpatient procedures and therefore some ECVs may be missed in these inpatient admission data. We were unable identify ineligibility for ECV based on fetal hypoxia, nuchal cord, and hyperextension of the fetal head. However these factors are unlikely to rule out a large proportion of women we have deemed eligible for ECV. Further, these and most of the other eligibility criteria are considered relative rather than absolute contraindications for ECV, suggesting our classification of eligibility for ECV is conservative.

Our definition of ECV success was based on presentation at delivery and this may result in some misclassification: a small proportion (3–5%) of pregnancies with immediately successful ECVs may revert back to breech presentation and be misclassified as “unsuccessful ECVs”. A similar proportion of unsuccessful ECVs may be misclassified as successful ECVs because the fetus has turned spontaneously [20]. This is unlikely to be problematic, given that ultimate ECV success may be viewed as achieving vaginal delivery.

We did not have information on the numbers of women who were offered and declined ECV or who experienced complications and adverse events associated with ECV. Improved monitoring and data collection via systematic recording of whether or not ECV was offered, declined, and attempted would provide valuable information on the population coverage of ECV and barriers to its uptake. Population-based information on the characteristics of ECV procedures, such as operator experience, use of tocolysis, adverse events, as well as outcomes, would provide valuable data that could contribute to maintaining and improving the quality of ECV services.

## Conclusions

Currently the majority of women with breech presentation in NSW do not undergo ECV, although greater uptake of ECV would reduce breech presentation, diminish the associated risks and costs of caesarean section and bypass those associated with vaginal breech birth. Improved monitoring of ECV attempts, success, and outcomes will aid in identifying barriers to ECV uptake, while specialised breech services, especially those targeted to women birthing in regional areas and private hospitals, and further training or capacity building among clinicians may facilitate greater use of ECV.

## Abbreviations

ECV: external cephalic version
NICU: neonatal intensive care unit
NSW: New South Wales, a state of Australia
SCN: special care nursery
